# Recombination-aware alignment of diploid individuals

**DOI:** 10.1186/1471-2164-15-S6-S15

**Published:** 2014-10-17

**Authors:** Veli Mäkinen, Daniel Valenzuela

**Affiliations:** 1 Helsinki Institute for Information Technology HIIT, Department of Computer Science, University of Helsinki, P.O. Box 68 (Gustaf Hällströmin katu 2b), 00014 Helsinki, Finland

**Keywords:** Alignment of labeled directed acyclic graphs, Variation calling evaluation, Haplotype assembly

## Abstract

**Background:**

Traditionally biological similarity search has been studied under the abstraction of a single string to represent each genome. The more realistic representation of diploid genomes, with two strings defining the genome, has so far been largely omitted in this context. With the development of sequencing techniques and better phasing routines through haplotype assembly algorithms, we are not far from the situation when individual diploid genomes could be represented in their full complexity with a pair-wise alignment defining the genome.

**Results:**

We propose a generalization of global alignment that is designed to measure similarity between phased predictions of individual diploid genomes. This generalization takes into account that individual diploid genomes evolve through a mutation and recombination process, and that predictions may be erroneous in both dimensions. Even though our model is generic, we focus on the case where one wants to measure only the similarity of genome content allowing free recombination. This results into efficient algorithms for direct application in (i) evaluation of variation calling predictions and (ii) progressive multiple alignments based on labeled directed acyclic graphs (DAGs) to represent profiles. The latter may be of more general interest, in connection to covering alignment of DAGs. Extensions of our model and algorithms can be foreseen to have applications in evaluating phasing algorithms, as well as more fundamental role in phasing child genome based on parent genomes.

## Introduction

A *diploid genome *consists of chromosome pairs, where one sequence of a pair is obtained from the mother and the other from the father, through a *recombination process*: The two sequences representing mother (or father) chromosome pair are mixed together into one sequence by copying large chunks alternatively from both copies. Mutations can occur so that the child chromosome pair is not an exact copy of recombined sequences inherited from the mother and the father.

The problem we tackle in this paper is how to define optimal alignment between two diploid chromosome pairs (called simply diploid genomes in the sequel), so that only the sequence content is taken into account allowing arbitrary recombinations.

To our surprise, this fundamental notion of sequence similarity has not been addressed before in the literature. This is apparently due to the strong role of the abstraction of one consensus string to represent genomes. This abstraction is sufficient in most comparative genomics scenarios when the aim is to measure large-scale mutation events for inter-species comparison, resulting into measures such as the *inversion distance *[1], the *inversion-indel distance *[2], and *DCJ-indel distance *[3,4], to name a few.

More fine-grained measures are needed when comparing individuals from the same species. Global alignment of individual consensus genomes only takes into account homozygous variants and one allele of each heterozygous variant. An obvious improvement is to model a diploid genome as a pair-wise alignment and to define a global alignment of two such pair-wise alignments. Such generalization is in the core of *progressive multiple alignment*, where alignment of alignments has been widely studied. However, most literature on progressive alignments abstracts partial multiple alignments as strings of columns, and just redefines the substitution operations as a measure of similarity between columns, e.g. as sum-of-pairs of possible character substitutions or as relative entropy of frequency profiles. String of columns is yet another abstraction of the underlying structure, and there has been huge amount of work to overcome its "once a gap, always a gap" shortcoming. Quite recently, approaches based on representing the underlying structure truthfully as a *node-labeled directed acyclic graph (DAG) *have been proposed to overcome this problem [5,6]. There, the core problem is to find a path *A *(represented as a string) through one DAG, and a path *X *through the other DAG, such that the optimal alignment of *A *and *X *has maximum score over all possible pairs of such paths. We refer to this problem as *path-alignment *on labeled DAGs. Notice that on the two DAGs created from two pair-wise alignments representing diploid genomes, *A *and *X *are sequences resulting from an arbitrary recombination of the underlying genomes.

We propose an extension of the DAG path-alignment considered in [5,6], in the special case of pair-wise alignments. Instead of extracting one path from each DAG, we extract two paths from each, forming a *covering alignment*: Let *G*^1 ^and *G*^2 ^be two labeled DAGs each representing a pair-wise alignment. We aim to find two paths *A *and *B *that cover all nodes of *G*^1^, and two paths *X *and *Y *that cover all nodes of *G*^2^, such that *S*(*A, X*)+ *S*(*B, Y*) is maximum over all path-covers of *G*^1 ^and *G*^2^, where *S*(·, ·) is the global alignment score. This approach takes into account *all *sequence content of diploid genomes (represented by DAGs *G*^1 ^and *G*^2^) for the similarity measure.

Unfortunately, we are not yet able to solve the general statement of the covering alignment problem. We solve a one-sided covering alignment problem, and a restricted covering alignment problem where the solution paths need to be synchronized. Both of them define a distance between diploid genomes. The latter problem admits a scalable solution in *O*(*DN*) time, where *D *is the resulting synchronized diploid to diploid edit distance and *N *the maximum diploid length. Experiments show that this approach can identify planted mutations accurately, indicating that the sacrifice in restricting the general problem statement appears not to be vital.

For simplicity of exposition, we describe our models and algorithms using (unit cost) edit distance in place of similarity score *S*(·, ·); however, our results can be trivially modified to compute the global alignment similarity measure. For the same reason, we derive our equations assuming free recombination; however, it is not difficult to include a cost of recombination in our equations.

### Applications

While we believe that covering alignment is a fundamental notion justified to be studied on its own rights, some direct applications follow: The more general one is as a generic tool for computing similarity between diploid genomes, taking into account the possibility of recombination (possibly including a penalty for recombination operations). Another application that follows from the problem statement, is that covering alignment can be plugged into progressive multiple alignment to take into account full information of the labeled DAGs instead of the partial information as in [5,6].

#### Variant calling evaluation

Another application is possible in *variant calling evaluation *[7], as covering alignment takes heterozygous variations properly into account: High-throughput sequencing allows a cost-effective way to discover how an individual genome differs from the consensus reference genome of the species. The result of such variant calling process is a list of homozygous and heterozygous variant predictions. To evaluate how good such prediction methods are, one can resort to simulating artificial diploid genome by applying a (random) set of variants to the reference. Let such simulated genome be called ground-truth and the included variants be called *ground-truth variants*. One can then simulate the sequencing of random DNA fragment reads from the ground-truth, to feed them to the variant calling method. The method will then infer the probable variants (typically by aligning the reads to the reference genome). Let these variants be called *predicted variants*.

Direct comparison of ground-truth and predicted variants is problematic due to invariant indels and prediction inaccuracies. Because of that, in [7] all predicted variants are applied to the reference in order to create a *predicted haploid genome*. Edit distance between the predicted haploid and the ground-truth diploid was then computed, allowing arbitrary recombinations for the haploid to distribute along the diploid, giving a distance measure. It was shown that this haploid to diploid edit distance can be computed on realistic size variant calling scenarios [7]. This approach is actually highly similar to the path-alignments of [5,6].

There remained one shortcoming in [7] due to the asymmetric measure; if there are overlapping predicted heterozygous variants, one needs to decide which ones to take to the predicted haploid. It was proposed to create another haploid with the remaining predictions that could not be applied in the first round, and do another haploid to diploid distance computation. However, such scheme is not fully rigorous as it favors sensitivity over specificity. Our new notion resolves the above issue.

#### Haplotyping

Finally, we believe that there is a more fundamental notion combining one-sided covering alignment and path-alignment that can be used in phasing child genome through mother-father-child trios: Conclusions section sketches how our one-sided covering alignment solution can be extended for this scenario.

## Methods

### Pair-wise alignment and edit distance

Let *A *and *B *be two sequences of size *N *and *M *respectively, over an alphabet ∑. A *pair-wise alignment *of sequences *A *and *B *is a pair of sequences (*S^A^, S^B^*) such that *S^A ^*is a supersequence of *A, S^B ^*is a supersequence of *B*, |*S^A^*| = |*S^B^*| = *L *is the length of the alignment, and all positions which are not part of subsequence *A *(respectively B) in *S^A ^*(respectively *S^B^*), contain the symbol '−'. The symbol '−' is a special character not in ∑.

Given a cost function *C *such that *C*(*a, b*) is the cost of transforming a character *a *into *b*, for any *a, b *∈ ∑ ∪ {'−'}, the cost of an alignment is defined as follows: $\text{C}\left(S^{A},S^{B}\right)=\sum_{i=1}^{L}C\left(S_{i}^{A},S_{i}^{B}\right)$. Typically, for any *a, b *∈ ∑ it holds that *C*(*a, b*) = *C*(*b, a*) > 0, *C*(*a, a*) = 0, and *C*(*a*, '−') = *C*('−', *a*) = *C_indel _*> 0 is the cost of inserting (or deleting) a character.

The *edit distance D*(*A, B*) between *A *and *B *can now be defined as follows: *D*(*A, B*) = *min*{C(*S^A^, S^B^*):(*S^A^, S^B^*) is an alignment of *A *and *B*}. An alignment that has the minimum cost is called an optimal alignment.

It is possible to compute the edit distance using dynamic programming in time *O*(*NM*), however, it is possible to reduce the running time further to *O*(*ND*), where *D *= *D*(*A, B*) is the *unit cost (Levenshtein) edit distance *[8,9].

### Diploid to diploid alignment

We propose a distance D((*A, B*), (*X, Y*)) to measure a distance between diploid individuals (*A, B*) and (*X, Y*) that allows them to recombine freely.

A recombination *R*(*S^A^, S^B^*) of an alignment (*S^A^, S^B^*) is another alignment $\left(S^{A^{\prime }},S^{B^{\prime }}\right)$ of some sequences *A*' and *B*' such that there exists a bitvector *I *such that $S^{A^{\prime }}\left[i\right]=S^{A}\left[i\right]$ and $S^{B^{\prime }}\left[i\right]=S^{B}\left[i\right]$ if *I*[*i*] = 1, and $S^{A^{\prime }}\left[i\right]=S^{B}\left[i\right]$ and $S^{B^{\prime }}\left[i\right]=S^{A}\left[i\right]$ if *I*[*i*] = 0.

**Diploid to diploid distance: **Given two pair-wise alignments (*S^A^, S^B^*) and (*S^X^, S^Y^*) the diploid to diploid distance is defined as

$\text{D}\left((A,B\right),\left(X,Y)\right)=min\{D\left(A^{\prime },X^{\prime }\right)+D\left(B^{\prime },Y^{\prime }\right):+\left(S^{A^{\prime }},S^{B^{\prime }}\right)$ is a recombination of (*S^A^, S^B^*) and $\left(S^{X^{\prime }},S^{Y^{\prime }}\right)$ is a recombination of (*S^X^, S^Y^*).

This problem can be interpreted as a covering alignment of two labeled DAGs created from pair-wise alignments: see the discussion in the end of synchronized diploid to diploid alignment section. Unfortunately, we do not know how to solve this problem efficiently or how to argue about its complexity. We leave this as an interesting open problem and continue to the variants that we know how to solve and that also have important applications; we also believe the basic notions derived for the variants give insights for tackling the general case later.

### Pair of haploids to diploid alignment

In this section we propose a distance D((*A, B*), *X, Y*) to measure a distance between diploid individual (*A, B*) and a pair of haploid sequences *X *and *Y*.

**Pair of haploids to diploid distance: **Given a pair-wise alignment (*S^A^, S^B^*) and two sequences *X *and *Y*, the pair of haploids to diploid distance is defined as $D\left((A,B),\left(X,Y\right)\right)=\text{min}\{D\left(A^{\prime },X\right)+D\left(B^{\prime },Y\right):\left(S^{A^{\prime }},S^{B^{\prime }}\right)$ is a recombination of (*S^A^, S^B^*)}.

**Figure 1:**
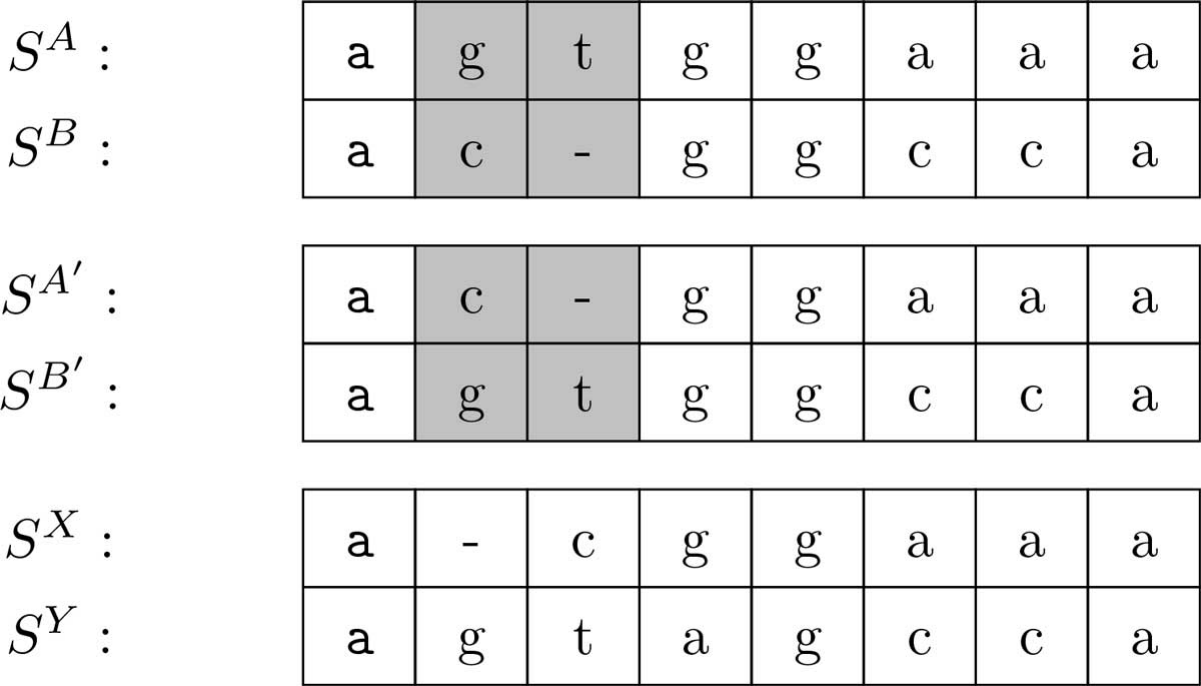
**A pair-wise alignment (*S^A^, S^B^*) is shown for sequences *A *= *agtggaaa *and *B *= *acggcca***. A recombination $S^{A^{\prime }}$ and $S^{B^{\prime }}$ is obtained by interchanging the shaded areas. The corresponding bitvector is *I *= 0110000. A pair-wise alignment (*S^X^, S^Y^*) is shown for sequences *X *= *acggaaa *and *Y *= *agtggcca*. The pair of haploids to diploid distance between (*S^A^, S^B^*) and (*X, Y*) is 0 + 1 = 1, and is obtained using the recombination $\left(S^{A^{\prime }},S^{B^{\prime }}\right)$ and unit cost edit distance as the measure. The plain unit cost edit distance between (*A, B*) and (*X, Y*) is 2 + 3 = 5. The plain unit cost edit distance between (*A, B*) and (*Y, X*) is 3 + 2 = 5. The synchronized diploid to diploid distance between (*S^A^, S^B^*) and (*S^X^, S^Y^*) is 3, using unit cost edit distance as the measure.

An example of recombination in pair-wise alignments and the pair of haploids to diploid distance measure is included in Figure 1. The motivation to study this measure is that the dynamic programming solution given below shows how the one-side covering alignment is computed. This prepares for the solution of the synchronized diploid to diploid distance studied in the sequel. More importantly, the Conclusions section describes an extension for modeling mother-father-child trios such that this cubic algorithm is only minimally modified.

#### A cubic time algorithm

To compute the pair of haploids to diploid distance, we propose to compute values *V*_*i,j,k *_for *i *∈ {1, . . . , *N*}, *j *∈ {1, . . . *M*}, and *k *∈ {1, . . . , *L*}, such that *V*_*i,j,k *_stands for the pair of haploids to diploid distance between sequences *X*_1*..i*_, *Y*_1*..j *_and alignment $\left(S_{1..k}^{A},S_{1..k}^{B}\right)$

For the sake of simplicity we first show the recurrence when *S^A^*[*k*] = *S^B^*[*k*] = *c *∈ ∑:

(1.1) $$V_{i,j,k}=min\{C\left(X_{i},c\right)+C\left(Y_{j},c\right)+V_{i-1,j-1,k-1},$$

(1.2) $$C(\prime -\prime ,c)+C(Y_{j},c)+V_{i,j-1,k-1},$$

(1.3) $$C(X_{i},c)+C(\prime -\prime ,c)+V_{i-1,j,k-1},$$

(1.4) $$C(\prime -\prime ,c)+C(\prime -\prime ,c)+V_{i,j,k-1},$$

(1.5) $$C\left(X_{i}{,}^{\prime }{-}^{\prime }\right)+V_{i-1,j,k},$$

(1.6) $$C(Y_{j},\prime -\prime )+V_{i,j-1,k}\}$$

Here, expression (1.1) stands for a match or substitution; expressions (1.2) to (1.4) stand for insertions either into *X, Y*, or both; and expressions (1.5) and (1.6) stand for deletion from *X *or *Y *. Note that we do not include the case $V_{i,j,k}=C\left(X_{i}{,}^{\prime }{-}^{\prime }\right)+C\left(Y_{i}{,}^{\prime }{-}^{\prime }\right)+V_{i-i,j-1,k}$ because it is always redundant: this case stands for a deletion from both *X *and *Y *at the same time, and that can be obtained by first deleting a character from *X*, and then from *Y*.

When *a *= S*^A^*[*k*] ≠ S*^B^*[*k*] = *b w*e have to consider symmetric cases:

(2.1) $$V_{i,j,k}=min\{C(X_{i},a)+C(Y_{j},b)+V_{i-1,j-1,k-1},$$

(2.2) $$C\left(X_{i},b\right)+C\left(Y_{j},a\right)+V_{i-1,j-1,k-1},$$

(2.3) $$C(\prime -\prime ,a)+C(Y_{j},b)+V_{i,j-1,k-1},$$

(2.4) $$C(X_{i},a)+C(\prime -\prime ,b)+V_{i-1,j,k-1},$$

(2.5) $$C(\prime -\prime ,a)+C(\prime -\prime ,b)+V_{i,j,k-1},$$

(2.6) $$C(\prime -\prime ,b)+C(Y_{j},a)+V_{i,j-1,k-1},$$

(2.7) $$C(X_{i},b)+C(\prime -\prime ,a)+V_{i-1,j,k-1},$$

(2.8) $$C(\prime -\prime ,b)+C(\prime -\prime ,a)+V_{i,j,k-1},$$

(2.9) $$C\left(X_{i}{,}^{\prime }{-}^{\prime }\right)+V_{i-1,j,k},$$

(2.10) $$C(Y_{j},\prime -\prime )+V_{i,j-1,k}\}$$

We need to consider twice as many expressions for matches/substitutions (2.1 and 2.2), twice as many conditions for insertions (2.3 to 2.8), and the same number of conditions for deletions (2.9 and 2.10). When *a *= *b *those duplicated equations become redundant and we recover the previous formulation. Also it would be possible to formulate simplified recursions when there is a gap in the alignment (that is, either *a *or *b *equals '−'), however those are also particular cases of the general formulation, given that *C*('−', '−') = 0.

To compute the recombinant distance using dynamic programming we need to fill a table *V *in time *O*(*MNL*). If we want to find the actual alignment, in addition to the standard traceback process, we need to traceback the table carrying a bitvector *I *which is initially empty. Every time we take a transition that decreases *k *we need to prepend a 0 (respectively a 1) to *I*, if we choose an expression from 2.1, 2.3, 2.4, or 2.5 (respectively 2.2, 2.6, 2.7, or 2.8). With this bitvector *I *we identify the recombination $\left(S^{A^{\prime }},S^{B^{\prime }}\right)$ that generated the optimal alignment, signaling with a 1 the positions where a recombination is done.

### Synchronized diploid to diploid alignment

Now let us assume that we have again two diploid genomes represented as pair-wise alignments. We derive a restricted variant of diploid to diploid alignment that keeps both input alignments *synchronized*. We first present some auxiliary concepts that are useful for expressing the algorithms.

#### Guiding functions

Given an alignment (*S^X^, S^Y^*) for the sequences *X *and *Y*, we build a *guiding function h *as follows: *h*(*z*) = (*h_i_*(*z*), *h_j_*(*z*)) is such that $h_{i}\left(z\right)=z-\text{count}\left(S_{1..z}^{X}{,}^{\prime }{-}^{\prime }\right)$ and $h_{j}\left(z\right)=z-\text{count}\left(S_{1..z}^{Y}{,}^{\prime }{-}^{\prime }\right)$.

**Figure 2:**
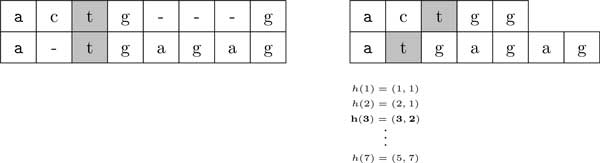
**For the sequences *X *= *actgg *and *Y *= *atgagag *we show a pairwise alignment composed by the sequences *S*^X ^= *actg *- - - *g*, and *S^Y ^*= *a *- *tgagag***. On the right the pairwise alignment is represented by the function *h*.

Given the two sequences *X *and *Y *and the guiding function *h*, it is straightforward to recover the alignment (*S^X^, S^Y^*). Therefore, a guiding function is an alternative representation of an alignment. The guiding function allows us to traverse *X *and *Y *in a way that both *h_i _*and *h_j _*increase in the areas of the alignment without gaps. In the gapped areas, only the index corresponding to the non-gapped sequence increases. Figure 2 shows an example.

Let us denote by $X_{1..i}|S_{1..k}^{X}$ and $Y_{1..j}|S_{1..k}^{Y}$ the longest prefixes of *X *and *Y *appearing inside the partial alignment $\left(S_{1..k}^{X},S_{1..k}^{Y}\right)$.

We say that two alignments $\left(S^{A^{\prime }},S^{X}\right)$ and $\left(S^{B^{\prime }},S^{Y}\right)$ are *synchronized *with alignment (*S^X^, S^Y^*) iff for each partial alignment $\left(S_{1..k}^{X},S_{1..k}^{Y}\right)$ with $X_{1..i}|S_{1..k}^{X}$ and $Y_{1..j}|S_{1..k}^{Y}$ there are partial alignments $\left(S_{1..p}^{A^{\prime }},S_{1..P}^{X}\right)$ and $\left(S_{1..q}^{B^{\prime }},S_{1..q}^{Y}\right)$ with $X_{1..i}|S_{1..p}^{X}$ and $Y_{1..j}|S_{1..q}^{Y}$ such that *p *= *h_i_*(*k*) and *q *= *h_j_*(*k*), where *h *is the guiding function of (*S^X^, S^Y^*).

**Synchronized diploid to diploid distance: **Given two pair-wise alignments (*S^X^, S^Y^*) and (*S^A^, S^B^*) of length *L*^1 ^and *L*^2 ^respectively, the synchronized diploid to diploid distance is defined as *min*{*D*(*A*', *X*)+ *D*(*B*', *Y*): $\left(S^{A^{\prime }},S^{B^{\prime }}\right)$ is a recombination of (*S^A^, S^B^*), and *D*(*A*', *X*) and *D*(*B*', *Y*) correspond to alignments that are synchronized with (*S^X^, S^Y^*)}.

**Figure 3:**
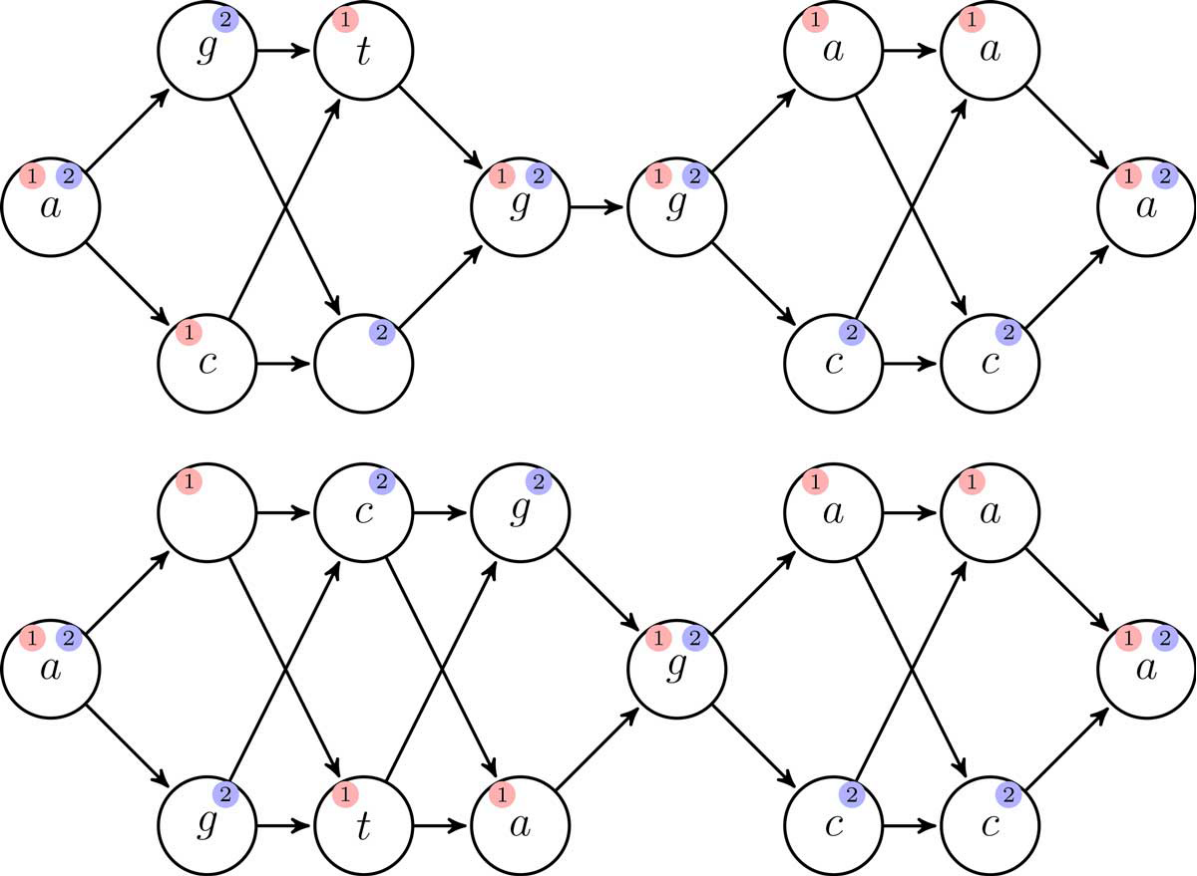
**DAG interpretation of the diploid to diploid alignment**. Above, a DAG that represents the alignment (*S*^A^, *S*^B^), and below, a DAG that represents the alignment (*S^X^, S^Y^*), both from the example of Figure 1. From each DAG we extract two covering paths. That means that every node is visited by at least one path. An optimal solution to the synchronized diploid to diploid distance is given by the two paths extracted from the first DAG generating the sequences *actggaaa *and *agggcca *and the two paths from the second one generating the sequences *atagaaa *and *agcggcca*. The synchronized diploid to diploid distance is then *d*(*actgaaa, ataaaa*) + *d*(*aggcca, agcgcca*) = 2 + 1 = 3. Here the unit cost edit distance scores are used. Observe that the optimal diploid to diploid distance is 1 corresponding to the solution of pair of haploids to diploid alignment in Figure 1, and that is also possible to extract paths that represent the solution of the diploid to diploid distance.

Figure 3 shows an example where the connection with the covering alignment problem is discussed.

The example in Figure 3 shows that synchronized diploid to diploid alignment is not giving optimal answer to the more general diploid to diploid alignment problem. The same example indicates that sometimes one can obtain the optimal answer through the pair of haploids to diploid alignment instead, but the following counterexample shows that this is not always the case: Consider the pair-wise alignments *S^A ^*= *tc, S^B ^*= *ag *and *S^X ^*= *ngc, S^Y ^*= *atv*. If the cost of any substitution is 10, and the cost of any indel is 1, the optimal diploid to diploid alignment is given by recombining the second alignment in the second position, by $d=D\left(S^{A},S^{X^{\prime }}\right)+D\left(S^{B},S^{Y^{\prime }}\right)=D\left(tc,ntc\right)+D\left(ag,agv\right)=2$. The optimal solution of the pair of haploids to diploid alignment problem, that allow recombinations only in the first pairwise alignment is *d *= *D*(*tc, ngc*)+ *D*(*ag, atv*) = 6.

#### A quadratic time algorithm

Now we can formalize a recurrence for the synchronized diploid to diploid alignment problem. The idea is that instead of varying *i *and *j *freely as in the cubic time algorithm, we will vary only the parameter of the guiding function, so that the guiding function provide us indexes *i *and *j *that are synchronized with the alignment (*S^X^, S^Y^*)

We compute values *D*_*z,k *_for *z *∈ {1, . . . , *L*^1^}, *k *∈ {1, . . . *L*^2^}, where *D*_*z,k *_stands for the synchronized diploid to diploid distance between sequences $X_{1..h_{i}\left(z\right)},Y_{1..h_{j}\left(z\right)}$ and alignment $\left(S_{1..k}^{A},S_{1..k}^{B}\right)$.

As the indexes *h_i_*(*z*) and *h_j_*(*z*) are given by the guiding function of (*S^X^, S^Y^*), the alignment is synchronized with the alignment (*S^X^, S^Y^*).

(3.1) $$D_{z,k}=min\{D_{z-1,k-1}+\zeta \left(h_{i}\left(z-1\right),h_{j}\left(z-1\right),k-1,h_{i}\left(z\right),h_{j}\left(z\right),k\right),$$

(3.2) $$D_{z,k-1}+\zeta \left(h_{i}\left(z\right),h_{j}\left(z\right),k-1,h_{i}\left(z\right),h_{j}\left(z\right),k\right),$$

(3.3) $$D_{z-1,k}+\zeta \left(h_{i}\left(z-1\right),h_{j}\left(z-1\right),k,h_{i}\left(z\right),h_{j}\left(z\right),k\right)\;\}$$

The rationale is to consider, among all the possibles transitions used in the cubic time algorithm for pair of haploids to diploid alignment, those that are compatible with the guiding function. In this way the alignment of *X *and *Y *is preserved. For this sake, the function *ζ *receives as input the triplet (*i, j, k*) that corresponds to the previous cell, and the triplet (*i*', *j*', *k*') that corresponds to the current cell.

Given that at least one of *z *or *k *decreases in each recursive call, the valid cases for the function *ζ *are those where at least one of *k, i, j *increase. For any of those cases, the valid recombination costs from equation 2 are considered:

The cases where both *k *and *z *increase are

(4.1) $$\zeta \left(i-1,j-1,k-1,i,j,k\right)=min\{C\left(X_{i},A_{k}\right)+C\left(Y_{j},B_{k}\right),$$

(4.2) $$C\left(X_{i},B_{k}\right)+C\left(Y_{j},A_{k}\right)\}$$

(5.1) $$\zeta \left(i,j-1,k-1,i,j,k\right)=min\{C(\prime -\prime ,A_{k})+C\left(Y_{j},B_{k}\right),$$

(5.2) $$C(\prime -\prime ,B_{k})+C(Y_{j},A_{k})\}$$

(6.1) $$\zeta \left(i-1,j,k-1,i,j,k\right)=min\{C\left(X_{i},A_{k}\right)+C(\prime -\prime ,B_{k}),$$

(6.2) $$C\left(X_{i},B_{k}\right)+C(\prime -\prime ,A_{k})\}$$

(7.1) $$\zeta \left(i-1,j,k-1,i,j,k\right)=min\{C\left(X_{i},A_{k}\right)+C(\prime -\prime B_{k}),$$

(7.2) $$C\left(X_{i},B_{k}\right)+C(\prime -\prime ,A_{k})\}$$

The cases where only *z *increases:

(8.1) $$\zeta \left(i-1,j-1,k,i,j,k\right)=C\left(X_{i}{,}^{\prime }{-}^{\prime }\right)+C\left(Y_{j}{,}^{\prime }{-}^{\prime }\right)$$

(8.2) $$\zeta \left(i,j-1,k,i,j,k\right)=C\left(Y_{j}{,}^{\prime }{-}^{\prime }\right)$$

(8.3) $$\zeta \left(i-1,j,k,i,j,k\right)=C\left(X_{i}{,}^{\prime }{-}^{\prime }\right)$$

The case where only *k *increases:

(9.1) $$\zeta \left(i,j,k-1,i,j,k\right)=C(\prime -\prime ,A_{k})+C(\prime -\prime ,B_{k})$$

#### An O(*ND*) time algorithm

The same technique [8,9] used to achieve *O*(*ND*) running time for the Levenshtein distance of strings can be applied here. The key observation here is

**Lemma ***Let *(*S^A^, S^B^*) *and *(*S^X^, S^Y^*) *be two alignments, and let D*_*z,k *_*be the synchronized diploid to distance costs computed as before. Then *∀*k *∈ 0, . . . , *L*^1^, *z *∈ 0, . . . *L*^2 ^*it holds D*_*z,k *_>*D*_*z*-1,*k *_and D_*z,k *_>*D*_*z,k*-1_.

*Proof *It is enough to verify that expressions 8.1 to 8.3, and 9.1 are always positive. This holds because *X *and *Y *contain no gaps. On the other hand, *S^A^*[*k*] or *S^B^*[*k*] might contain a gap, but it is not possible that both of them contain a gap at the same time.   □

Let us assume for simplicity of exposition that |*L*^1^| = |*L*^2^| = *N *and we store values *D_z_*_,*k *_in a table. We want to test if the distance between (*A, B*) and (*X, U*) is smaller than a threshold *t *or not. That is, we want to test if *D_N_*_,*N *_≤ *t *for some threshold *t*. To do that, we do not need to fill the entire table. It is enough to consider the diagonals *j *- *i *∈ {-*t/*2, -*t/*2 + 1, . . . , 0, 1, . . . , *t/*2} in the computation, because any path using a diagonal outside this *zone *must use more than *t *operations that have a positive cost, leading to an alignment with cost greater than *t*. Starting with *t *= 1 and doubling this value until *D_N_*_,*N *_does not decrease any more gives the optimal answer, and the final area where the computation is done is of order *O*(*ND*); the previous zone sizes form a geometric series, so the computation done inside them is of the same order as the computation inside the final zone.

## Results and discussion

We implemented the three algorithms in *C*++. For the computation of the Levenshtein distance, we resorted to a boost-compatible implementation of a *O*(*ND*) time algorithm [9]. We ran our experiments in a computer node with 2 Intel Xeon E5540 2.53GHz processors, 32GB of RAM. The operating system was Ubuntu 12.04.4. Our code was compiled with gcc 4.6.4, optimization option -*O*3.

Our first experiments compared the performance of our three algorithms. In order to do that, we considered samples from the human chromosome 21 of different sizes between 1000 and 100000. In order to generate the pair of diploid individuals we made random mutations (SNPs and deletions) independently on four copies of the sequence. The pair of haploids for the cubic time algorithm were extracted from one of the generated diploids. Table 1 shows the results. As expected, the cubic and quadratic time algorithms became infeasible for moderate size sequences.

**Table 1:** The distance between pairs of diploids computed by our three algorithms, and the time (in seconds) used for the computation.

		Cubic algorithm		Synchronized -Matrix		Synchronized -Diagonals	
**Length**	**Mutations**	**Distance**	**Time**	**Distance**	**Time**	**Distance**	**Time**

1000	21	21	19.190	21	0.040	21	0.001
2000	47	43	154.030	47	0.110	47	0.010
4000	85	79	1219.060	85	0.650	85	0.020
8000	174	-	-	172	1.790	172	0.070
10000	218	-	-	216	2.820	216	0.180
20000	408	-	-	403	11.080	403	0.580
40000	777	-	-	750	44.290	750	1.170
80000	1578	-	-	1531	177.200	1531	4.630
100000	1994	-	-	1935	276.960	1935	11.500

Cubic algorithm is our implementation of the pair of haploids to diploid algorithm, Synchronized -Matrix is the straightforward implementation of the synchronized diploid to diploid distance, and Synchronized -Diagonals is the *O*(*ND*) time implementation of the same algorithm using the shortest detour technique.

Then we wanted to test our new formulations on more realistic instances. We simulated again a pair of diploid individuals that contained the same variants, but this time they were recombined differently. In addition we added mutations independently in every strand. The results are shown in Table 2. Our synchronized diploid to diploid distance correctly identifies the planted mutations. This indicates that the synchronization does not make the general diploid to diploid distance overly restricted.

**Table 2:** A number of variations were applied to the reference genome, and blocks of size 200 were recombined freely.

			Synchronized diploid To diploid		Levenshtein	
**Length**	**Variations**	**Mutations**	**Distance**	**Time**	**Distance**	**Time**

10000	100	7	7	0.006	52	0.010
20000	227	18	18	0.020	139	0.052
30000	338	33	33	0.064	241	0.138
40000	434	36	36	0.096	272	0.214
50000	538	46	46	0.140	367	0.362
60000	637	50	50	0.230	448	0.528
70000	742	67	67	0.274	529	0.850
80000	839	73	73	0.370	562	1.066
90000	960	91	91	0.550	697	1.570
100000	1089	109	109	0.688	762	2.198
1000000	10127	990	990	58.978	7676	376.320

After that, random mutations were introduced with probability 0.001. We show the distances and times (in seconds) obtained by our synchronized diploid to diploid distance algorithm and by the Levenshtein distance.

Same kind of diploid genomes can also result from variation calling predictions after applying haplotype assembly [10]; long haplotype blocks can be correctly phased, but at regular intervals, low read coverage results into phasing errors with the order of the top and bottom sequences switched when compared to the simulated ground-truth.

## Conclusions

We proposed new metrics to compare diploid individuals, extending classical pair-wise sequence alignment, to compare a pair of pair-wise alignments under free recombination. Our motivation for the study came from variant calling evaluation, where one wants to ignore the phasing errors. For some other applications, one might want to add a penalty on the recombination events. Such penalties are easy to add to the formalism and to the algorithms. A prominent application for such an approach is the evaluation of phasing algorithms: The optimal covering alignment also defines the recombination positions, so one could compare two alignments resulting from different phasing algorithms and evaluate how close they are, without requiring a ground-truth. Comparison against ground-truth gives an estimate on how many phasing errors the predicted diploid contains. One could actually apply our current model for this application, but free recombinations may give too much freedom to optimize sequence content so that predictions could seem to be containing many phasing errors.

We want to emphasize that our model captures the similarity between predicted diploid genomes, and if we want to use it for modelling the actual recombinations appearing during evolution some modifications are required: The reason is that full information of child diploid genome comes from parts of mother and father diploid genomes. The partial measure in terms of path-alignment [5,6] captures this kind of one-to-one ancestral comparison: Let G*^m^*, G*^f^*, A, and *B denot*e a labeled DAG representing mother, a labeled DAG representing father, and two haploid sequences representing a child diploid genome, respectively. We find a path *X i*n G*^m ^*and a path *Y i*n G*^f ^*such that max(*S*(*X, A*) + *S*(*Y, B*), *S*(*Y, A*) + *S*(*X, B*)) is maximum, where *S*(·, ·) is the optimal alignment score for two sequences. The mutations contributing to the optimal solution give a way to trace the mutations coming from mother (father) lineage. Observe that, before phasing, our information on the child genome is as well just a labeled DAG G*^c ^*created from a pair-wise alignment representation of predicted diploid. To find the optimal phasing of child genome, we could try to find (*X, Y*) trough a one-sided covering alignment of G*^c ^*to the path-alignments in G*^m ^*and G*^f^*. This is almost identical to our pair of haploids to diploid alignment, except in the place of pair of haploids we have a pair of diploids represented by labeled DAGs. The cubic time algorithm extends to this case: Topological traversal of G*^m ^*and G*^f ^*in place of two sequences results into some more recurrence options to take into account the in-neighbors for each pair of nodes (*v^m^, v^f^*) from the two DAGs. We find this an extremely promising approach to tackle the phasing problem and as future work we will conduct experiments on simulated models. In the same line of development, we will try to make this approach scalable by exploiting similar properties as those used for the shortest detour speed-up.

Finally, although the restricted variant of diploid to diploid alignment using synchronization results into good experimental behaviour, studying the complexity of the general diploid to diploid alignment is certainly another focus for our future work.

## Competing interests

The authors declare that they have no competing interests.

## Authors' contributions

Both authors contributed equally to the development of the models and algorithms, as well as to the writing of this manuscript. DV implemented the algorithms and performed the experiments.

## Declarations

Publication costs for this article were funded by by the Finnish Center of Excellence in Cancer Genetics Research.

This article has been published as part of *BMC Genomics *Volume 15 Supplement 6, 2014: Proceedings of the Twelfth Annual Research in Computational Molecular Biology (RECOMB) Satellite Workshop on Comparative Genomics. The full contents of the supplement are available online at http://www.biomedcentral.com/bmcgenomics/supplements/15/S6.

## References

[B1] Hannenhalli S Pevzner PA Transforming cabbage into turnip: polynomial algorithm for sorting signed permutations by reversals Journal of the ACM (JACM) 1999 46 1 1 27 10.1145/300515.300516

[B2] Willing E Zaccaria S Braga MDV Stoye J On the inversion-indel distance BMC Bioinformatics 2013 14 S15 S3 10.1186/1471-2105-14-S15-S3 24564182 PMC3851949

[B3] Yancopoulos S Attie O Friedberg R Efficient sorting of genomic permutations by translocation, inversion and block interchange Bioinformatics 2005 21 16 3340 3346 10.1093/bioinformatics/bti535 15951307

[B4] Braga MDV Willing E Stoye J Genomic distance with dcj and indels Algorithms in Bioinformatics (WABI) 2010 90 101

[B5] Lee C Grasso C Sharlow MF Multiple sequence alignment using partial order graphs Bioinformatics 2002 18 452 64 10.1093/bioinformatics/18.3.452 11934745

[B6] Löytynoja A Vilella AJ Goldman N Accurate extension of multiple sequence alignments using a phylogeny-aware graph algorithm Bioinformatics 2012 28 1684 1691 10.1093/bioinformatics/bts198 22531217 PMC3381962

[B7] Mäkinen V Rahkola J Haploid to diploid alignment for variation calling assessment BMC Bioinformatics 2013 14 Suppl 15 13 Presented at RECOMB-CG 10.1186/1471-2105-14-S15-S13 24564537 PMC3852041

[B8] Ukkonen E Algorithms for approximate string matching Information and control 1985 64 1 100 118 10.1016/S0019-9958(85)80046-2

[B9] Myers EW An *O*(*ND*) difference algorithm and its variations Algorithmica 1986 1 1-4 251 266 10.1007/BF01840446

[B10] Patterson M Marschall T Pisanti N van Iersel L Stougie L Klau GW Schönhuth A Whatshap: Haplotype assembly for future-generation sequencing reads18th Annual International Conference on Research in Computational Molecular Biology (RECOMB 2014) Lecture Notes in Computer Science 2014 8394 237 249

